# Analysis of the Single-Cell Heterogeneity of Adenocarcinoma Cell Lines and the Investigation of Intratumor Heterogeneity Reveals the Expression of Transmembrane Protein 45A (TMEM45A) in Lung Adenocarcinoma Cancer Patients

**DOI:** 10.3390/cancers14010144

**Published:** 2021-12-29

**Authors:** Patrícia Neuperger, József Á. Balog, László Tiszlavicz, József Furák, Nikolett Gémes, Edit Kotogány, Klára Szalontai, László G. Puskás, Gábor J. Szebeni

**Affiliations:** 1 Laboratory of Functional Genomics, Biological Research Centre, Temesvári krt. 62, H6726 Szeged, Hungary; neuperger.patricia@brc.hu (P.N.); balog.jozsef@brc.hu (J.Á.B.); gemes.nikolett@brc.hu (N.G.); kotogany.edit@brc.hu (E.K.); 2 Ph.D. School in Biology, University of Szeged, H6726 Szeged, Hungary; 3 Department of Pathology, University of Szeged, Állomás u. 2, H6725 Szeged, Hungary; tiszlavicz.laszlo@med.u-szeged.hu; 4 Department of Surgery, University of Szeged, Semmelweis u. 8., H6725 Szeged, Hungary; furak.jozsef@med.u-szeged.hu; 5 Csongrád County Hospital of Chest Diseases, Alkotmány u. 36., H6772 Szeged, Hungary; szalontai@deszkikorhaz.hu; 6 Avicor Ltd., Alsó kikötő sor 11/D, H6726 Szeged, Hungary; 7 Department of Physiology, Anatomy and Neuroscience, Faculty of Science and Informatics, University of Szeged, Közép fasor 52, H6726 Szeged, Hungary; 8 CS-Smartlab Devices, Ady E. u. 14., H7761 Kozármisleny, Hungary

**Keywords:** non-small cell lung cancer, adenocarcinoma, intra-cell-line heterogeneity, intratumor heterogeneity, TMEM45A

## Abstract

**Simple Summary:**

Non-small cell lung cancer (NSCLC) is one of the main causes of cancer-related deaths worldwide. Intratumoral heterogeneity (ITH) is responsible for the majority of difficulties encountered in the treatment of lung-cancer patients. Therefore, the heterogeneity of NSCLC cell lines and primary lung adenocarcinoma was investigated by single-cell mass cytometry (CyTOF). Human NSCLC adenocarcinoma cells A549, H1975, and H1650 were studied at single-cell resolution for the expression pattern of 13 markers: GLUT1, MCT4, CA9, TMEM45A, CD66, CD274, CD24, CD326, pan-keratin, TRA-1-60, galectin-3, galectin-1, and EGFR. The intra- and inter-cell-line heterogeneity of A549, H1975, and H1650 cells were demonstrated through hypoxic modeling. Additionally, human primary lung adenocarcinoma, and non-involved healthy lung tissue were homogenized to prepare a single-cell suspension for CyTOF analysis. The single-cell heterogeneity was confirmed using unsupervised viSNE and FlowSOM analysis. Our results also show, for the first time, that TMEM45A is expressed in lung adenocarcinoma.

**Abstract:**

Intratumoral heterogeneity (ITH) is responsible for the majority of difficulties encountered in the treatment of lung-cancer patients. Therefore, the heterogeneity of NSCLC cell lines and primary lung adenocarcinoma was investigated by single-cell mass cytometry (CyTOF). First, we studied the single-cell heterogeneity of frequent NSCLC adenocarcinoma models, such as A549, H1975, and H1650. The intra- and inter-cell-line single-cell heterogeneity is represented in the expression patterns of 13 markers—namely GLUT1, MCT4, CA9, TMEM45A, CD66, CD274 (PD-L1), CD24, CD326 (EpCAM), pan-keratin, TRA-1-60, galectin-3, galectin-1, and EGFR. The qRT-PCR and CyTOF analyses revealed that a hypoxic microenvironment and altered metabolism may influence cell-line heterogeneity. Additionally, human primary lung adenocarcinoma and non-involved healthy lung tissue biopsies were homogenized to prepare a single-cell suspension for CyTOF analysis. The CyTOF showed the ITH of human primary lung adenocarcinoma for 14 markers; particularly, the higher expressions of GLUT1, MCT4, CA9, TMEM45A, and CD66 were associated with the lung-tumor tissue. Our single-cell results are the first to demonstrate TMEM45A expression in human lung adenocarcinoma, which was verified by immunohistochemistry.

## 1. Introduction

Lung cancer (LC) is the most frequent cancer, comprising 22–23% of all cases [1]. The heterogeneity of LC among patients, such as intertumoral or intralesional heterogeneity, was discovered quite early by pathologists, and these morphological types are small cell lung carcinoma (15% of all LC) and non-small cell lung carcinoma (NSCLC, 85% of all LC). NSCLC, in the traditional histologic classification, includes adenocarcinoma (40% of all LC), squamous cell carcinoma (30–35% of all LC), and pulmonary large-cell neuroendocrine carcinoma (3–5% of all LC) [2]. Our study is focused on NSCLC patients that had a poor prognosis of approximately 15–20% five-year overall survival [3]. Therapeutic failure, such as acquired resistance to chemotherapy or immunotherapy, relies on the clonal evolution of resistant tumor cells [4]. Clonal heterogeneity has remained undetected up to now because previous studies focused on the bulk mass of tumor cells while analyzing overall parameters, which overlooked the versatile phenotypical plasticity of the individual component cells or minor populations [5]. Recent molecular analysis has shown that the different genetic driver mutations in patients with greater population diversity, mainly those with adenocarcinoma, comprised a larger number of different subtypes [6]. The sequencing of 3243 patient-derived human adenocarcinoma tumor tissues revealed the following mutations along with their incidence: *EGFR* (55.9%), *KRAS* (11.7%), *NRAS* (0.7%), *PIK3CA* (2.9%), *HER2* (2.1%), *BRAF* (1.6%); fusions of *ALK* (2.8%), *ROS1* (0.6%), *RET* (0.6%), and amplifications in *MET* (1.3%) [7]. Deep-sequencing studies have identified the genomic landscape of NSCLC with 40% monoclonal and 60% polyclonal tumor evolution exhibiting temporal tumor heterogeneity [3,8]. Another level of diversity is the spatial intratumoral heterogeneity (ITH) of malignant cells. However, the occurrence of multilevel heterogeneity in lung adenocarcinoma was revealed as a result of innovations in methods and their sensitivity during the era of single-cell genomics and single-cell proteomics in tumor biology [9]. It was shown that karyotype heterogeneity, a clonal diversity in the genome, endows cancers cells with different growth potentials, where the outliers offer alternative routes for evolution within the same tumor [10]. In addition, metabolic heterogeneity has also been shown to be an obstacle of targeted LC therapy, as enhanced glycolysis supplies the energy for the main tumor mass, but different regions of the same tumor that use lactate as a carbon source were identified in human NSCLC patients [11]. However, we are closer to deciphering ITH through both genetic and molecular profiling, but the regulatory pathways governing ITH and the role of TME, including immune infiltrates, remain to be characterized. However, somatic mutations, genomic instability, and epigenetic control responding to the variety of external conditions, such as hypoxia, have been revealed as mechanisms that underlie ITH [12,13]. Because of the phenotypical plasticity and biological diversity of LC, proper histologic and biomarker profiling—as well as the characterization of its subtypes—are necessary for specialized treatment and prognosis [14,15]. ITH is predominantly responsible for relapses after therapy due to the therapeutic resistance achieved by certain tumor cell clones or a number of the tumor cell clones being located in a zone with limited circulation [16]. Therefore, further understanding of ITH, together with the development of novel drugs and delivery systems to mitigate its impact, is highly anticipated [17].

To address these goals, the repertoire of model systems in cancer research laboratories includes cell lines, three-dimensional cultures such as different cell-line organoids, murine syngeneic tumor models, and xenografts [18]. Notably, culturing cell lines in different laboratories has been shown to result in the evolution of inter-cell-line heterogeneity across laboratories, as was recently reported in a comprehensive genomic analysis of the 27 laboratory versions of MCF-7 human breast cancer cell lines [19] or the 14 laboratory versions of HeLa cells [20]. However, cell lines represent the easiest and most cost-efficient cancer biology models that can represent the arsenal of biochemical pathways, the structural and functional organelles of live cells, and drug-target expression. Other advantages of cell lines are that they can be scaled up for high-throughput screening systems and, in addition, can be genetically modified. The transcriptional intra-cell-line heterogeneity of the SUM197 breast cancer cell line [21] or LC2 adenocarcinoma cell line was recently studied by single-cell RNA sequencing [22]. The existence of subclones of human A549 adenocarcinoma cells with different sensitivity to actinomycin-D has been known of for some time [23]. Korsnes et al. followed the single-cell tracking of A549 cells upon yessotoxin treatment and created drug-response pedigree trajectories [24]. These studies suggested the intra-cell-line heterogeneity of A549 adenocarcinoma cells but were lacking protein-marker profiling at single-cell resolution.

In this study, we focused on the deciphering of intra-and inter-cell-line heterogeneity of A549, H1975, and H1650 human lung adenocarcinoma cell lines. The A549 cells are EGFR and cMET wild-type but with KRAS mutants (G12S); the H1975 cells are EGFR mutants (L858R, T790M) and cMET and KRAS wild-type; the H1650 cells are EGFR mutants (deletion E746, A750) and cMET and KRAS wild-type [25]. We also investigated the ITH of human primary lung adenocarcinoma, the most prevalent LC, by single-cell mass cytometric (CyTOF) immunophenotyping [9]. In their seminal paper, Lavin et al. reported the complexity of the immune infiltrate in early adenocarcinoma using CyTOF [26]. Therefore, our interest turned toward the marker profiling of malignant cells. Previously, we showed that A549 cells in three-dimensional cultures, as multicellular in vitro cellular models, were between the two-dimensional cell cultures and ex vivo xenograft models in terms of cell-surface-expression patterns of the investigated 12 proteins [27]. Namely, GLUT1 (solute carrier family 2, facilitated glucose-transporter member 1), MCT4 (monocarboxylate transporter 4), CA9 (carbonic anhydrase 9), TMEM45A, CD66 (carcinoembryonic antigen-related cell adhesion molecules), CD24 (signal transducer CD24), and EGFR (epidermal growth factor receptor) targets were selected by differential expression in xenografts versus a two-dimensional cell culture of A549 cells analyzed using a 624 gene-based human cancer panel [27]. Five additional proteins were also studied in our previous work due to their relevant functions in adenocarcinoma tumor progression and therapy resistance [27], such as carcinoma stem-cell markers: TRA-1-60 [28], CD326 (epithelial cell adhesion molecule) [29], galectin-3 (Gal-3) [30], the immune checkpoint molecule CD274 (programmed cell death ligand-1) [31], and carcinoma marker cytokeratins (pan-keratin) [32]. We have made significant contributions in deciphering the role of the immunoregulator galectin-1 (Gal-1) in tumor-immune escape mechanisms [33,34,35,36]. Therefore, anti-galectin-1 antibody was added to our current study in addition to the previously established lung adenocarcinoma CyTOF panel.

In our current work, we use the single-cell mass cytometry adenocarcinoma panel to investigate (i) the intra- and inter-cell-line heterogeneity of three human adenocarcinoma cell lines, A549, H1975, and H1650; (ii) adenocarcinoma cell line heterogeneity by mimicking a hypoxic environment; (iii) ITH and TMEM45A expression in human primary lung adenocarcinoma versus corresponding healthy human lung tissue used as a control.

In this study, we show that A549, H1975, and H1650 NSCLC adenocarcinoma cell lines were not represented by uniform cells but, rather, bear intrinsic clonal heterogeneity for the 13 measured proteins. Additionally, we analyzed the ITH of human primary lung adenocarcinoma for 14 markers and are the first to report TMEM45A expression in lung adenocarcinoma.

## 2. Results

### 2.1. Clonal Heterogeneity of NSCLC Cell Lines

**Figure 1:**
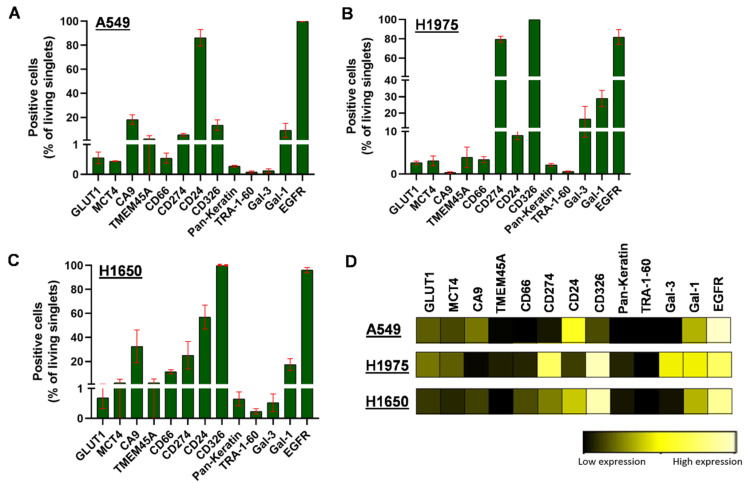
The intra-cell-line heterogeneity of A549, H1975, and H1650 human NSCLC cells analyzed by single-cell mass cytometry. Column bars of the (**A**) A549, (**B**) H1975, and (**C**) H1650 cells show the percentages of live single cells expressing the given cell-surface protein markers. Thirteen protein markers—GLUT1, MCT4, CA9, TMEM45A, CD66, CD274, CD24, CD326, pan-keratin, TRA-1-60, Gal-3, Gal-1, and EGFR—were investigated with single-cell resolution provided by the metal-tag-labeled antibodies used for mass cytometry. (**D**) Heatmap of the expression intensities of the 13 protein markers, which were calculated from the transformed ratio of medians of the table minimum using X-axis channels: panel/channel values.

We investigated the heterogeneity of A549, H1975, and H1650 human NSCLC adenocarcinoma subtype cell lines as most of the scientific achievements in cancer biology and drug discovery have involved cell lines. First, we verified the absence of any cross-contamination, which has been reported as a hidden problem in research laboratories worldwide [37]. The authentication of the cell lines was performed by profiling of short tandem repeat loci (STRs) (Supplementary Materials Tables S1–S3). The clonal heterogeneity of A549, H1975, and H1650 NSCLC cells was determined by the quantitation of the percentages of single cells positive for GLUT1, MCT4, CA9, TMEM45A, CD66, CD274, CD24, CD326, pan-keratin, TRA-1-60, Gal-3, Gal-1, and EGFR. The percentages of populations positive for a given marker delineated the heterogeneous pattern of the protein expression profile of A549 (Figure 1A), H1975 (Figure 1B), and H1650 cells (Figure 1C). The population percentages demonstrated intra- and inter-cell-line heterogeneity of A549, H1975, and H1650 human NSCLC adenocarcinoma cell lines (Figure 1A–C).

Since population percentages do not represent the extent of protein expression intensity, heatmaps were used to demonstrate pairwise comparisons of the expression intensities (Figure 1D). Median metal intensities proportional to the protein densities of single cells correspond to the extent of expression of the studied 13 markers and show heterogenous expression of the three cell lines. The heatmap shows the inter-cell-line heterogeneity of the adenocarcinoma cells while the following markers showed the highest expression in the panel: EGFR and CD24 in the case of A549; CD326 and CD274 for H1975; and CD326 and EGFR for H1650 (Figure 1D).

**Figure 2:**
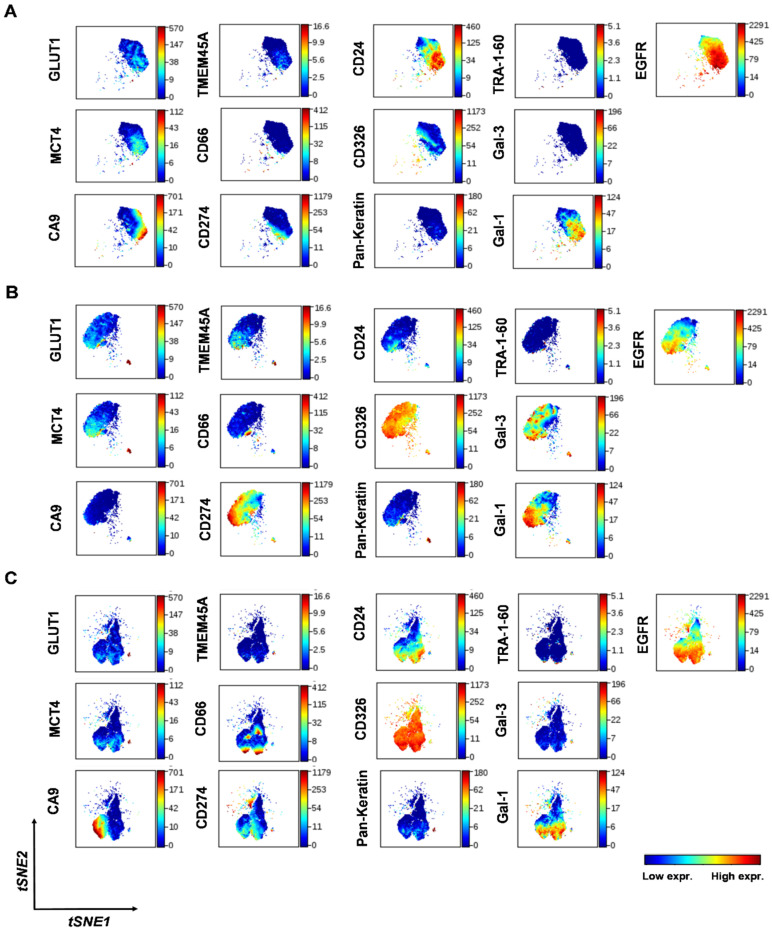
Single-cell heterogeneity of the human NSCLC cell lines. Representative multidimensional visualization of t-distributed stochastic neighbor embedding (viSNE) analysis of 13 protein markers at single-cell resolution in (**A**) A549, (**B**) H1975, and (**C**) H1650 cells. The analysis was performed within 10^5^ cells (iterations = 2000, perplexity = 30, theta = 0.5).

In order to visualize the clonal subpopulations of NSCLC cells, t-distributed stochastic neighbor embedding (t-SNE) analysis was performed on A549, H1975, and H1650 cells in one round [38], which mapped cells in a 13-dimensional space based on their expression of the studied 13 markers at single-cell resolution (Figure 2). The morphologic segmentations of the t-SNE maps allow the visualization of sample heterogeneity, a higher resolution of cell mapping, and the determination of common markers that are more highly expressed. The unsupervised multidimensional comparison of the cell-surface-marker expression of A549 (Figure 2A), H1975 (Figure 2B), and H1650 (Figure 2C) single cells reveals both different populations within the cell lines, as well as inter-cell-line heterogeneity.

**Figure 3:**
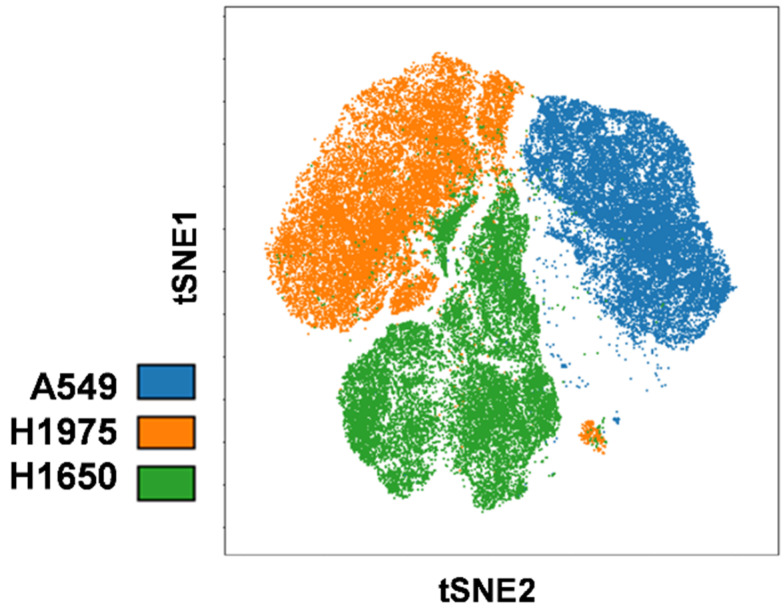
Inter-cell-line heterogeneity of the NSCLC cell lines. Merged viSNE plots of the three NSCLC cell lines in which 13 markers were simultaneously analyzed at single-cell resolution, with delineation of a characteristic map of A549, H1975, and H1650 cells.

In the visualization of intra-cell-line heterogeneity, the amount of coloration is proportional to the number of proteins detected on the cell surface. The coloration and their appearance in these viSNE plots demonstrate subpopulations based on the expression of characteristic markers in A549, H1975, and H1650 NSCLC cells (Figure 2). In merging the viSNE plots of the multiplex immunophenotyping data for simultaneous analysis 13 markers of single cells, the A549, H1975, and H1650 cells can be separated in multidimensional space with the delineation of three different ‘islands’, in which H1975 and H1650 partially overlap (Figure 3).

**Figure 4:**
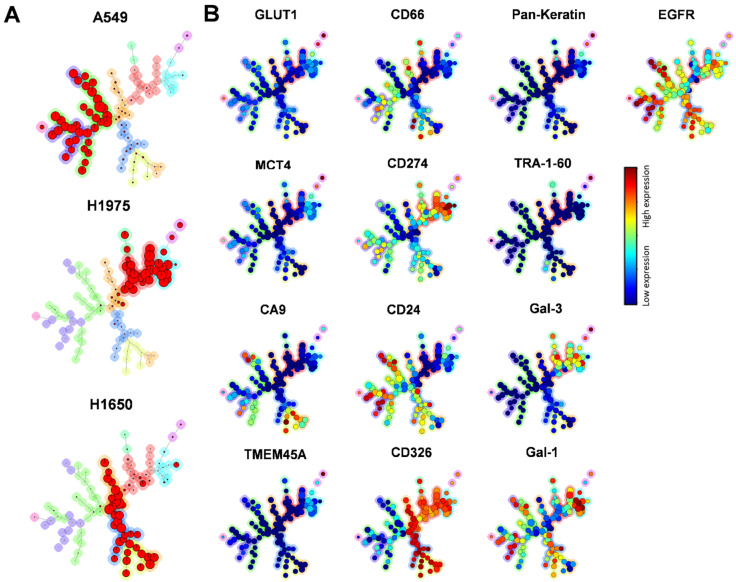
FlowSOM analysis of A549, H1975, and H1650 cells. (**A**) The lineages of tumor cell-line subpopulations were traced and visualized by unsupervised FlowSOM, an algorithm creating MSTs during automated, unsupervised clustering in Cytobank. The A549, H1975, and H1650 cells, based on their different marker-expression profiles, were plotted on different branches (red) of MSTs as cells with inter-cell-line heterogeneity (A549: left branch; H1975: right branch; H1650: lower branch). (**B**) Protein expressions of the three studied cell lines on the aggregated MSTs. Similar cells are assigned to the same node, and the size of node corresponds to the number of events within that cluster; the main subsets are highlighted in the graph. The expression intensities are indicated with coloration varying from blue (low expression) to red (high expression).

Intra-and inter-cell-line heterogeneity was further elucidated through the tracing and comparison of lineages of the subpopulations of A549, H1975, and H1650 cells by unsupervised flow data using a self-organizing map (FlowSOM) analysis (Figure 4) [39]. The three studied human adenocarcinoma cell lines were plotted to different branches (red) of the minimum spanning trees (MSTs) as cells with inter-cell-line heterogeneity in terms of different marker-expression profiles (Figure 4A). The populations of cells determined positive for a given marker were plotted on a nodule on the aggregated minimum spanning trees, where the coloration indicates the marker-expression intensity (Figure 4B)

### 2.2. Clonal Heterogeneity of NSCLC Cell Lines Is Preserved under Hypoxic Condition

**Figure 5:**
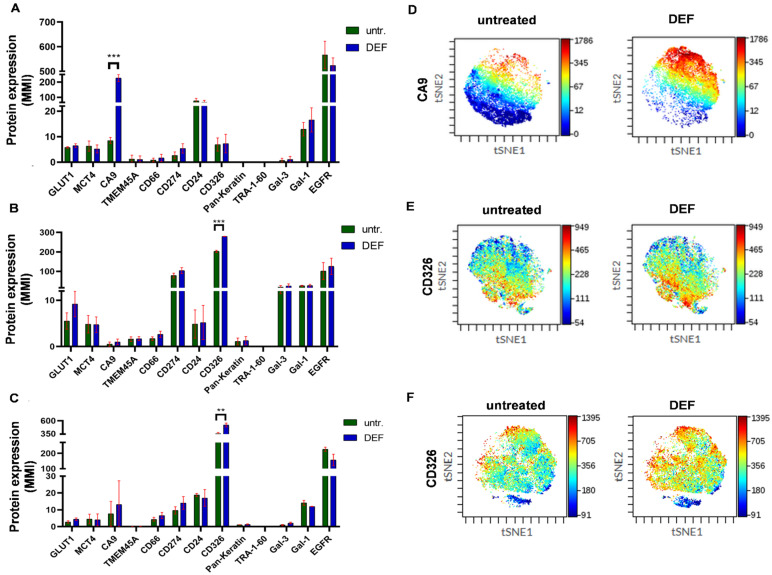
The heterogeneity of A549, H1975, and H1650 human NSCLC cells was preserved after mimicking hypoxia. Column bars of the (**A**) A549, (**B**) H1975, and (**C**) H1650 cells show the expression intensity (MMI = median metal intensity) of live single cells expressing the given cell surface protein markers after deferoxamine (DEF) treatment or when left untreated (untr.). Thirteen protein markers, GLUT1, MCT4, CA9, TMEM45A, CD66, CD274, CD24, CD326, pan-keratin, TRA-1-60, Gal-3, Gal-1, and EGFR, were investigated with single-cell resolution provided by the metal-tag-labeled antibodies used for mass cytometry. The viSNE pattern of the expression intensities shows an increase upon mimicking hypoxia of the (**D**) CA9 for A549 or CD326 for both (**E**) H1975 and (**F**) H1650. ** *p* < 0.01, *** *p* < 0.001.

The heterogeneity of A549, H1975, and H1650 cells was investigated after deferoxamine (DEF) treatment. Prolyl hydroxylase (PHD) requires iron as a cofactor, and upon normoxia, hydroxylation of hypoxia-inducible factor-1α (HIF-1α) is followed by polyubiquitination, which marks it for proteasomal degradation [40]. The hypoxic environment of the DEF model inhibits the activity of prolyl hydroxylase via iron chelation, which leads to HIF-1α stabilization [41]. The sublethal dose of DEF was determined by the resazurin viability assay with serial dilution of DEF (Supplementary Materials Figure S1). The effect of 400 µM DEF treatment was examined by the expression of genes with a hypoxia-responsive element (HRE) that are involved in angiogenesis (*VEGFA, PDGFB*), metabolism (*SLC2A1, LDHA, PGK1, PDK1*), metastasis (*CXCL12, CXCR4, PLAUR*), or acidosis (*LAMP2, CA9, PLAU*) and that of *TMEM45A* after 24 h incubation (Supplementary Materials Table S4). The induction of *TMEM45A* expression at a transcriptional level in hypoxia has previously been shown by others [42,43,44]. The quantitative real-time polymerase chain reaction (qRT-PCR) data verified the application of DEF for the establishment of the hypoxic model due to the upregulated transcription of known HIF-1α responsive genes (Supplementary Materials Figure S2). However, the A549, H1975, and H1650 adenocarcinoma cells showed different, cell-line characteristic gene expression profiles that further verified their biological diversity and the inter-cell-line heterogeneity of these NSCLC cell lines (Supplementary Materials Figure S2). Single-cell mass cytometry of the studied human adenocarcinoma cell lines also showed a cell-line characteristic profile of the tumor markers localized to the cell surface (Figure 5A–C). Mimicking hypoxia led to significant increase in only CA9 for A549 as well as CD326 for both H1975 and H1650 cells (Figure 5D–F). TMEM45A cell-surface-expression intensity remained low for A549, H1975, and H1650 monolayer cell cultures (Figure 5A–C).

### 2.3. Intratumor Heterogeneity of Primary Human Lung Adenocarcinoma

**Figure 6:**
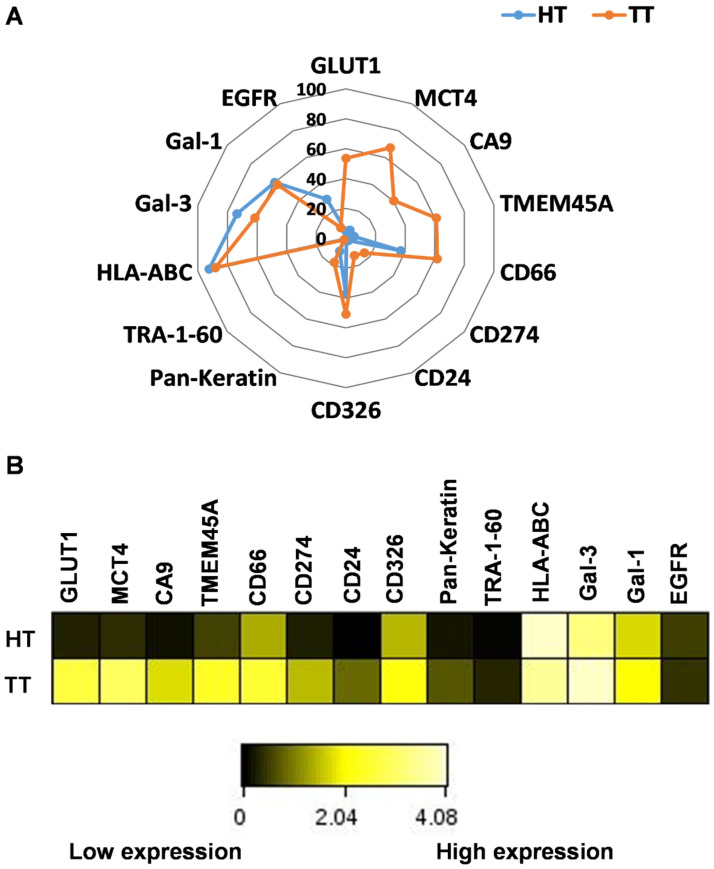
The pattern of the expression of the 14 proteins investigated in primary human lung adenocarcinoma cells. (**A**) Radar plot delineates the trajectories of the percentage of cells positive for the investigated markers. The higher positivity for GLUT1, MCT4, CA9, TMEM45A, CD66, CD274, CD24, CD326, and pan-keratin associated with TT versus HT. (**B**) Differences in the expression of carcinoma markers regarding protein density (median metal intensity) in single cells were visualized on a heatmap (calculated transformed ratio of medians by the table minimum).

We have shown that both intra-cell-line and inter-cell-line single-cell heterogeneity are present in the analyzed human lung adenocarcinoma cell lines, but whether this heterogeneity is inherited from the original tumor or acquired via genetic shift and microevolution of laboratory cell culturing is unknown. Therefore, we sought to address this by investigating a patient-derived primary lung adenocarcinoma sample freshly delivered to the laboratory after surgery. The nonconfidential clinical data of the patient (patient 1) can be found in Supplementary Materials Table S5. Both tumor tissue (TT) and a small piece of healthy control lung tissue (HT) was excised during the surgical intervention and processed for mass cytometry in our laboratory within 1 h. Single cells were gated on ^191^Ir+/^193^Ir+ DNA intercalator double-positive events, as previously described [27]. We focused on tumor cells; therefore, the immune infiltrate was excluded by gating on anti-human CD45− cells. The anti-HLA-ABC antibody was added to the panel, as diminished HLA-ABC expression can indicate the reduced immunosurveillance of tumors. The percentage of single cells positive for a given marker in TT vs. HT were the following: GLUT1 (53% vs. 4.8%), MCT4 (67.3% vs. 6.0%), CA9 (40.4% vs. 3.6%), TMEM45A (61.1% vs. 5.6%), CD66 (61.7% vs. 37.1%), CD274 (15.6% vs. 2.4%), CD24 (12.8% vs. 0.9%), CD326 (50.7 vs. 37.4%), pan-keratin (17.8% vs. 9.5%), TRA-1-60 (1.5% vs. 0.9%), HLA-ABC (88.1% vs. 92.4%), Gal-3 (61.5% vs. 73.6%), Gal-1 (57.7% vs. 60.1%), and EGFR (7.8% vs. 28.9%), respectively (Figure 6A). The positivity of cells for GLUT1, MCT4, CA9, TMEM45A, CD66, CD274, CD24, CD326, and pan-keratin were associated with TT, which allowed delineation—from those associated with HT—of tumor-specific trajectories on a radar plot.

**Figure 7:**
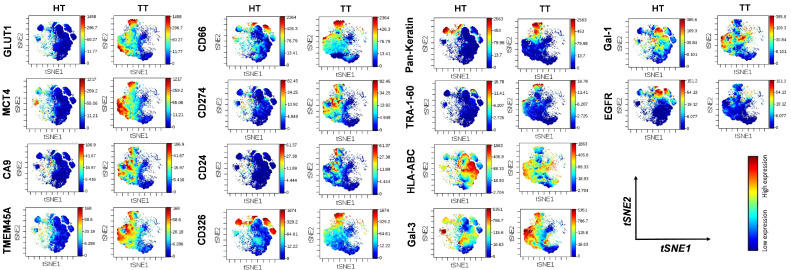
Single-cell intratumor heterogeneity of human primary lung adenocarcinoma. The viSNE analysis of single cells shows differential expression of GLUT1, MCT4, CA9, TMEM45A, CD66, CD274, CD24, CD326, pan-keratin, TRA-1-60, HLA-ABC, Gal-3, Gal-1, and EGFR in human primary lung adenocarcinoma. Mass cytometry revealed intratumor heterogeneity of human primary adenocarcinoma at protein level with single-cell resolution. The viSNE analysis displays the cell-surface-expression pattern of markers of single cells isolated from TT versus cells from HT of the same patient.

Protein expression intensities for GLUT1, MCT4, CA9, TMEM45A, CD66, CD274, CD24, CD326, and pan-keratin were also increased on the cell surface of cells liberated from tumor tissue versus non-involved healthy lung-tissue-derived cells, which was visualized as a heatmap (Figure 6B). Although the percentages of cells positive for Gal-3 or Gal-1 did not increase in TT vs. HT (Figure 6A), the expression intensity was brighter in TT vs. HT (Figure 6B). In line with the textbook explanation that tumor cells tend to downmodulate antigen presentation, the exposure of HLA-ABC on tumor cells decreased with the decrease in median metal intensity from 176 (TT) to 87 (HT). The intratumor heterogeneity was further dissected at the single-cell level using high-dimensional t-SNE analysis in Cytobank (Figure 7). The distribution of cells on the viSNE graphs demonstrated the clonal or diffuse expression of markers characteristic of the given sample. The coloration is proportional to the density (amount) of that protein on the cell surface (Figure 7).

**Figure 8:**
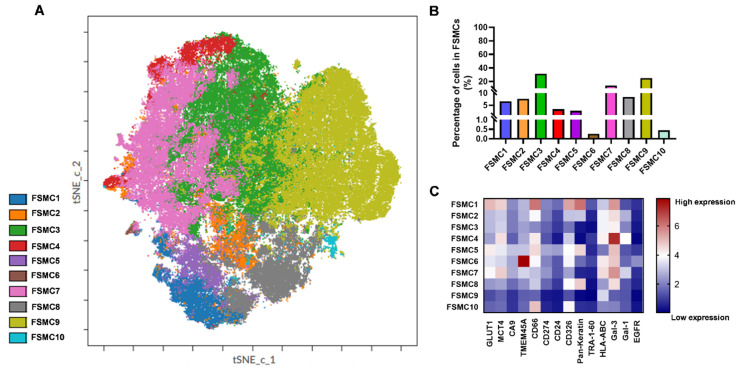
Unsupervised population analysis determined 10 FSMCs displayed on a viSNE map of the TT specimen. The distribution of FlowSOM metaclusters on the single-cell tSNE-CUDA plot (**A**). The percentage of cells in the FSMCs (**B**). Heatmap of the expression intensities of the measured 14 proteins in the FSMCs followed by arcsinh transformation (scale factor 5) (**C**).

Ten FlowSOM metaclusters (FSMCs) were identified when running FlowSOM on the t-SNE Compute Unified Device Architecture (CUDA) map as an unsupervised analysis in Cytobank. The FSMCs are populations of single cells plotted on a viSNE plot. These 10 FSMCs represent 10 distinct populations of cells isolated from the TT specimen (Figure 8).

Coexpression analysis was also carried out by manual gating on CD45− singlets (Supplementary Materials Figure S3A). These percentages of coexpressing cells were identified, which were not exclusive populations: CD326+/pan-keratin+ (15.6%); CD326+/GLUT1+ (6.9%); CD326+/MCT4+ (7.1%); CD326+/Gal-3+ (10.8%); CD326+/CD66+ (9.3%) (Supplementary Materials Figure S3B); CD66+/pan-keratin+ (12.1%); CD66+/GLUT1+ (8.8%); CD66+/MCT4+ (8.53%); CD66+/Gal-3+ (10.4%) (Supplementary Materials Figure S3C); Gal-3+/pan-keratin+ (13.9%); Gal-3+/GLUT1+ (19.2%); Gal-3+/MCT4+ (30.3%) (Supplementary Materials Figure S3D); MCT4+/pan-keratin+ (6.9%); MCT4+/GLUT1+ (16.6%) (Supplementary Materials Figure S3E); GLUT1+/pan-keratin+ (8.2%) (Supplementary Materials Figure S3F).

### 2.4. TMEM45A Expression in Primary Human Lung Adenocarcinoma

**Figure 9:**
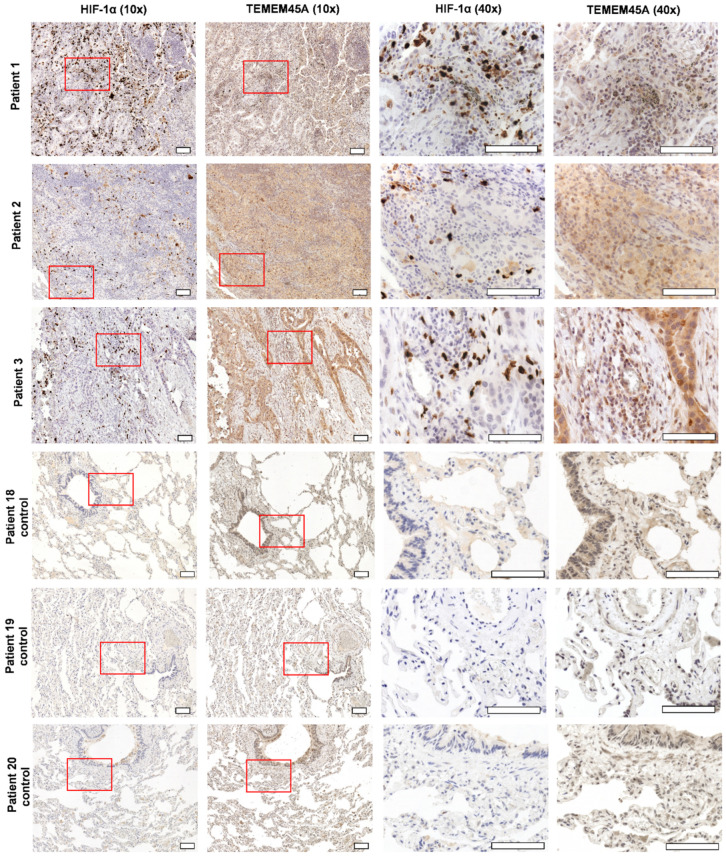
The immunohistochemistry showed high expression of TMEM45A and sporadic expression of HIF-1α in invasive acinar lung adenocarcinoma. An area of higher magnification (40× objective) within the presented image of the region of interest at lower magnification (10× objective) is indicated by a red box. Stages were the following: for patient 1, T1bN2M0; patient 2, T3N0M0; patient 3, T2bN2M0. Control tissues were derived from pneumothorax nontumorous patients. Scale bar: 100 µm.

Our interest then turned toward TMEM45A because its expression in primary human lung adenocarcinoma cells has not been previously described. Since obtaining both fresh human tissues of lung adenocarcinoma and of a non-involved adjacent tissue from the same patient is hard to achieve, we used immunohistochemistry (IHC) to investigate TMEM45A expression in 17 lung adenocarcinoma patients (patients 1–17) versus 3 nontumorous patients (patients 18–20) with pneumothorax (Figure 9 and Supplementary Materials Table S5). Consecutive sections of the formalin-fixed paraffin-embedded (FFPE) lung biopsies were stained with anti-HIF-1α or anti-TMEM45A antibodies.

Our report is the first to describe the expression of TMEM45A in human lung adenocarcinoma versus nontumorous lung tissue (Figure 9 and Supplementary Materials Figure S4). Although several authors have reported the expression of TMEM45A in hypoxia, most of these studies have relied on transcriptomics data [42,43,44]. Our IHC results showed the sporadic staining of HIF-1α (+) but prevalent TMEM45A staining (+++) in all 17 human lung adenocarcinoma tissues (Figure 9 and Supplementary Materials Figure S4).

## 3. Discussion

The ITH may be responsible for metastasis, acquired drug resistance, immune evasion and, finally, relapse [4,45]. Therefore, the degree of ITH has become a biomarker that is inversely proportional to patient prognosis [46]. A recent whole-genome sequencing study of 2658 tumor samples representing 38 cancer types underlined the selection of subclonal driver mutations behind ITH [47]. ITH may fundamentally originate from the variable genomic, transcriptomic, epigenetic, and proteomic states of single cells within a bulk tumor mass [48,49]. Wu et al. recently investigated 42 NSCLC patients by single-cell RNA sequencing and revealed their ITH by transcriptome analysis [50].

In our study, the state-of-the-art single-cell technology CyTOF was applied to decipher the intra-cell-line heterogeneity (ITH) of human primary lung adenocarcinoma at the protein level. First, we analyzed three human NSCLC cell lines of A549, H1975, and H1650 adenocarcinoma cells to determine whether these cell lines are represented by a mass of uniform cells or bear some characteristic cell-line heterogeneity. There are no previous reports in which single-cell results are available for these human lung adenocarcinoma cells measured at protein level. All three cell lines demonstrated single-cell heterogeneity for the 13 investigated markers and preserved their inherent clonal heterogeneity under hypoxia. We could not obtain CyTOF data from the original biopsies, from which the A549, H1975, and H1650 adenocarcinoma cells were derived, but we could show the intra- and inter-cell-line heterogenous single-cell-expression profiles of the 13 investigated markers GLUT1, MCT4, CA9, TMEM45A, CD66, CD274, CD24, CD326, pan-keratin, TRA-1-60, galectin-3, galectin-1, and EGFR. Therefore, our results confirm the validity of applying A549, H1975, and H1650 NSCLC cell lines as model systems for cancer research that focuses on cancer-cell heterogeneity. Hypoxia is one of the best-known driving forces of tumor microevolution, so NSCLC cells were treated with sublethal doses of DEF to mimic hypoxia. A known list of genes with HRE was assayed by qRT-PCR to validate the established hypoxic condition. Although qRT-PCR profiles of genes involved in angiogenesis (*VEGFA, PDGFB*), metabolism (*SLC2A1, LDHA, PGK1, PDK1*), metastasis (*CXCL12, CXCR4, PLAUR*), and acidosis (*LAMP2, CA9, PLAU*) as well as *TMEM45A* verifying the hypoxic effect of 400 µM DEF at the mRNA level, our CyTOF profile showed changes in only CA9 for A549 as well as in CD326 for both H1975 and H1650. Taken together, we could definitively show that the three different studied human lung adenocarcinoma cell lines—A549, H1975, and H1650—were not a mass of uniform cells; instead, these cell lines are represented by a heterogeneity of single cells and express cell-surface markers with different outcomes; they are, therefore, applicable in cancer studies focusing on cancer-cell heterogeneity.

Furthermore, the intratumor heterogeneity of human primary adenocarcinoma was investigated at the single-cell level by CyTOF. We showed the increased expression of GLUT1, MCT4, CA9, TMEM45A, and CD66, as compared to non-involved healthy lung tissue-derived single cells from the same patient. The GLUT1, MCT4, and CA9 are markers of metabolic adaptation to hypoxia and acidosis contributing to tumor aggressiveness, recurrence, and the poor prognosis of patients [51,52,53]. Taken together, targeting cancer metabolism in NSCLC has become a well-established concept [54], especially in targeting GLUT1, which facilitates glycolysis and promotes NSCLC via integrin β1/Src/FAK signaling [55]. The development of blocking agents for MCT4, which supports aerobic glycolysis via lactate efflux [56], and inhibitors of CA9—the master regulator of acidic extracellular pH during hypoxic adaptation—is under development [57]. CD66-a/c/e have been used as markers for epithelial tumor since 1965, as these proteins contribute to the proliferation, migration, and therapeutic resistance of carcinoma cells [58,59]. Previous single-cell proteomic studies have primarily focused on the immune compartment of lung adenocarcinoma [26,60]. Mistry et al. reviewed the advantages of CyTOF in studying solid tumors, namely for the quantitative analysis of certain proteins in single cells [61]. Using CyTOF, CD24 was previously used to grade ovarian cancer [62], cytokeratin was applied in labeling melanoma cells [63], and EpCAM was recently selected for labeling of ovarian carcinoma cells [64]. However, our study is the first to show the single-cell CyTOF profile of the non-immune compartment of human primary lung adenocarcinoma for the expression of the 14 investigated markers.

By understanding the single-cell marker-expression profiles, the ITH of lung cancer patients may reveal potential targets and support novel drug development and the design of personalized therapies [65]. In their seminal paper, Tavernari et al. showed the spatial heterogeneity of human adenocarcinoma samples, where the driving forces of ITH were found to be epigenetic and transcriptional reprogramming [66].

Our focus turned toward TMEM45A, as TMEM45A is the less-characterized protein among the others under investigation in our study. Little is known about the role of TMEM45A in NSCLC. However, it has been reported that the inhibition of TMEM45A may increase the chemosensitivity of cancer cells [67]. Others have shown the expression of the *TMEM45A* mRNA in hypoxia. Namely, Rendon et al. studied the expression of *TMEM45A* transcript in hypoxic CD133+ umbilical-cord blood cells [42]. Furthermore, Benita et al. identified *TMEM45A* as a HIF-1α target gene using in silico analysis [43]. Lastly, Flamant et al. described the expression of *TMEM45A* mRNA in hypoxic MDA-MB-231 human breast cancer cells and in HepG2 human hepatoma cells [44]. Hayez was the first to show the increase in the amount of TMEM45A protein close to the Golgi apparatus under hypoxic condition in murine embryonic fibroblasts [68]. In our IHC results, the expression of TMEM45A protein in lung adenocarcinoma was not exclusively reliant on HIF-α induction.

TMEM45A protein expression has been published in several cancer cell lines, such as MDA-MB-231 breast cancer cells and in HepG2 human hepatoma cells [44], and in several other cancers such as human cervical lesions [69], ovarian cancer [70], glioma [71,72], renal cell carcinoma [73,74,75], colorectal cancer [76], head and neck cancer [75], and invasive breast cancer [77]. In our previous work, we reported the increased cell-surface expression of TMEM45A in three-dimensional cultures of A549 cells and in murine A549 xenograft tumors by CyTOF [27]. Our current study is to first to report the increased cell-surface expression of TMEM45A protein in patient-derived lung adenocarcinoma samples using CyTOF. The expression of TMEM45A in lung adenocarcinoma was demonstrated through IHC of 17 patients. Although IHC may detect both cell surface and intracellular TMEM45A protein load, we speculated that cell surface expression of TMEM45A detected by CyTOF may be a conditional phenomenon, and the regulation of the trafficking and the function of TMEM45A warrants further research.

## 4. Materials and Methods

### 4.1. Cell Culturing

The human non-small cell lung cancer (NSCLC) cells, namely adenocarcinoma cell lines A549, H1975, and H1650, were purchased from the American Type Culture Collection. The H1975 and H1650 cells were maintained in DMEM or A549 cells in DMEM/F12 (DMEM, PAN-Biotech GMBH, Aidenbach, Germany; F12 Nut mix, Gibco, Thermo Fisher Scientific, Waltham, MA, USA) containing 4.5 g/L glucose, 10% fetal bovine serum (FBS) (Gibco), 2mM GlutaMAX (Gibco, Waltham, MA, USA), 100U/mL penicillin, and 100 µg/mL streptomycin antibiotics (penicillin G sodium salt and streptomycin sulfate salt, Sigma-Aldrich, St. Louis, MI, USA). The cells were cultured in a standard tissue culture Petri dish, 10 mm in diameter (Corning Life Sciences, Corning, NY, USA) at maximum 80% confluence in a standard atmosphere of 95% air and 5% CO_2_.

### 4.2. Cell Line Authentication

Pellet of 1 × 10^6^ cells was washed twice using PBS, dissolved in 0.5 mL 90% ethanol, and shipped to Microsynth AG (Balgach, Switzerland) for cell authentication. Profiling of human cell lines A549, H1975, and H1650 was completed using highly polymorphic short tandem repeat (STR) loci. STR loci were amplified using the PowerPlex 16 HS System (Promega, Madison, WI, USA). Fragment analysis was completed on an ABI3730xl (Life Technologies, Thermo Fisher Scientific, Waltham, MA, USA) and the resulting data were analyzed with GeneMarker HID software (Softgenetics, State Collage, PA, USA).

### 4.3. Cell Viability Assay

The viability of cells was determined by the fluorescent resazurin (Sigma-Aldrich, St. Louis, MI, USA) assay, as previously described [78]. Briefly, an aliquot of all types of cells (10,000/well) were seeded into 96-well plates (Corning Life Sciences, Corning, NY, USA) in 80 µL cell culture media. Deferoxamine (DEF) (Sigma-Aldrich) was dissolved in cell culture media in 10 mM. The stock solution of DEF was sterile-filtered (0.22 µm, low binding filter, Merck, Darmstadt, Germany). Serial dilutions of DEF were prepared in complete cell culture media: 5 mM, 2.5 mM, 1.25 mM, 625 µM, 312.5 µM, and 156.25 µM. Treatment with DEF was applied in 20 µL per well in duplicates. Final concentrations of DEF were as follows: 1000, 500, 250, 125, 62.5, and 31.25 µM. Resazurin reagent (Sigma-Aldrich) was dissolved in PBS (pH 7.4) at 0.15 mg/mL concentration, filtered through a 0.22 µm membrane, and aliquoted and stored at −20 °C. After 72 h incubation at 37 °C under 5% CO_2_ (Sanyo, Osaka, Japan), we applied resazurin 20 µL stock to 100 µL culture. After 2 h incubation at 37 °C under 5% CO_2_ (Sanyo), fluorescence (530 nm excitation/580 nm emission) was recorded on a multimode microplate reader (Cytofluor 4000, PerSeptive Biosytems, Framingham, MA, USA). Proliferation was calculated in relation to untreated cells, and all values were normalized to blank wells containing media without cells.

### 4.4. Quantitative Real-Time Polymerase Chain Reaction (qRT-PCR)

Cells were plated (2 × 10^6^/well) in 6-well plates (Corning Life Sciences) in 2 mL media followed by a 24 h resting period to allow time for the proper attachment of the cells. The DEF was dissolved in complete DMEM–F12 media in 400 µM, filtered using a low-binding 0.22 μm sterile filter (Merck, Darmstadt, Germany), and following the removal of the supernatant, 400 µM DEF was added to the treated wells in 2 mL media. After 24 h treatment, cells were washed with PBS and harvested in Accuzo (Bioneer, Daedeok-gu, Daejeon, Korea) and stored at −80 °C. The RNA was isolated, as described previously [79]. Briefly, RNA was isolated with the Direct-zol RNA Miniprep Kit (ZymoResearch, Irvine, CA, USA), according to the manufacturer’s instructions. The reverse transcription of 3 µg RNA to cDNA in 30 µL final volume was carried out using the High-Capacity cDNA Reverse Transcription Kit (Thermo Fisher Scientific), according to the manufacturer’s instructions. Reagents of the kit were diluted to 1× concentration based on the initial concentration as indicated: 10× reaction buffer, 10× random primer, 25× dNTP mix, and 20× reverse transcriptase. The transcription was performed in the MyGenie 32 Thermal Block (Bioneer, Daejeon, South Korea) according to the following protocol: 25 °C for 10 min, 37 °C for 120 min, 0 °C for 5 min, 75 °C for 10 min, and held at 8 °C. After reverse transcription, cDNA was diluted by adding 130 µL DNase-free water (Thermo Fisher Scientific). The quantitative real-time polymerase chain reaction (qRT-PCR) was carried out using the LightCycler 96 System (Roche, Basel, Switzerland), as previously described [80]. Briefly, the 10 µL qRT-PCR reactions contained 1 µL template cDNA, 250 nM gene specific primer pairs (Eurofins Genomics, Ebersberg, Germany), the 5 µL qPCRBIO SyGreen Mix (Byosystems, London, UK), and 3 µL ultrapure water. The primer sequences and corresponding accession numbers are listed in Supplementary Materials Table S4. The PCR protocol was as follows: enzyme activation at 95 °C for 2 min, 45 cycles of denaturation at 95 °C for 10 s, annealing at 60 °C, and extension at 60 °C for 10 s. All PCRs were performed with three replicates for each sample. After amplification, the melting curve was reviewed to verify the specificity of the PCR reactions. The cycle-threshold (Ct) values were normalized to the ACTB gene. Relative expressions of the analyzed genes were normalized to the mean value of the ACTB reference gene (ΔCt = Ct_gene_ − Ct_ACTB_), and gene expression changes were calculated as the average of three replicates. Data are expressed as ΔΔCt (log_2_) values and were normalized to expression values from cells maintained in normoxia: (ΔΔCt (log_2_) = ΔCt_normoxia_ − ΔCt_DEF_). All values were presented as mean ± standard deviation (SD). Data were visualized using GraphPad Prism 6 software (GraphPad Prism Software Inc., San Diego, CA, USA).

### 4.5. Ethical Statement

The subjects gave their informed consent for inclusion before participating in the study. The study was conducted in accordance with the Declaration of Helsinki, and the protocol was approved by the Ethics Committee of the University of Szeged under the 163/2018-SZTE Project identification code.

### 4.6. Human Lung Tissue Homogenization

Lung tissue was surgically removed and freshly transported to our laboratory in RPMI media at 4 °C. The non-involved lung (healthy tissue, HT) and tumor tissue (TT) were separated, minced by scissors and forceps aseptically into small pieces, and enzymatically digested by 250 µg/mL Liberase research-grade (Sigma-Aldrich), 100 µg/mL DNaseI (Sigma-Aldrich) in FCS-free RPMI for 60 min at 25 °C using a magnetic stirrer. Single-cell suspension was filtered through sterile gauze and 100 µm cell strainer (Merck). Following centrifugation (300 *g*, 5 min), red blood cells were lysed in 5 mL ACK solution (0.15 M NH_4_Cl, 10 mM KHCO_3_, and 0.1 mM Na_2_EDTA at pH 7.3, Molar Chemicals Ltd. Hungary) for 3 min. Following washing (in which 10 mL RPMI was added followed by centrifugation at 300× *g* for 5 min), cells were counted using a Bürker chamber with viability based on trypan blue dye (Sigma-Aldrich) staining for single-cell mass cytometry.

### 4.7. Single-Cell Mass Cytometry

Cell lines (A549, H1975, and H1650) were washed with PBS and detached using Accutase (Corning Life Sciences). Cells were counted using Bürker chamber with viability based on trypan blue dye staining. Three million cells pooled from three biological replicates of the cell lines were processed for mass cytometry staining in suspension in PBS, as previously described with some modifications [81]. Single-cell suspensions from healthy non-involved tissue (HT = healthy tissue) and malignant adenocarcinoma (TT = tumor tissue) were compared from one patient. Cell viability was determined by cisplatin (5 μM 195Pt, Fluidigm) staining for 3 min on ice in 300 μL PBS. The sample was diluted with 1500 μL Maxpar Cell Staining Buffer (MCSB, Fluidigm, San Francisco, CA, USA) and centrifuged at 350× *g* for 5 min. Cells were suspended in 50 μL MCSB, and the antibody mix was added in a volume of 50 μL. The list of the antibodies used for mass cytometry is in Table 1.

The following antibodies were conjugated with metal tags in house using the Maxpar metal labeling kit strictly according to the manufacturer’s instructions (Fluidigm, South San Fransisco, CA, USA): anti-HLA-ABC, anti-CA9, anti-GLUT1, anti-MCT4, anti-TMEM45A, and anti-Gal-1. Antibodies were titrated prior to the experiment to determine the optimal dilution. Cell lines A549, H1975, and H1650 were individually barcoded by labeling with HLA-ABC in the following combinations prior to incubation with the cocktail of antibodies for our markers of interest: untreated, HLA-ABC ^112^Cd; DEF-treated, HLA-ABC ^114^Cd. Three biological replicate experiments were performed for each cell line and each condition. Cell lines were incubated with the anti-HLA-ABC antibodies for live-cell barcoding at 4 °C for 30 min, then washed twice with 2 mL MCSB and with centrifugation at 300× *g* for 5 min. The barcoded cells were pooled into separate tubes for A549, H1975, and H1650. HLA-ABC ^144^Nd was used to label liberated cells from the primary human lung tissue.

After 60 min incubation with the cocktail of the antibodies at 4 °C, antibodies were washed twice with 2 mL MCSB and with centrifugation at 300× *g* for 5 min. The pellet was suspended in the residual volume. Cells were fixed in 1.6% formaldehyde (freshly diluted from 16% Pierce formaldehyde with PBS, Thermo Fisher Scientific) and incubated for 10 min at room temperature. Cells were centrifuged at 800× *g* for 5 min. Cell ID DNA intercalator (^191^Ir and ^193^Ir, Fluidigm) diluted 1000× into Maxpar Fix and Perm (Fluidigm) was added and incubated overnight at 4 °C. Cells for the acquisition were centrifuged at 800× *g* for 5 min and then were washed using 2 mL MCSB and centrifuged at 800× *g* for 5 min. Cells were suspended in 1 mL PBS (for WB injector, Fluidigm) and counted with a Bürker chamber during centrifugation. For the acquisition, the concentration of cells was set to 0.5 × 10^6^/mL in cell-acquisition solution (CAS) (Fluidig) containing 10% EQ calibration beads. Cells were filtered through 30 μm gravity filter (Celltrics Sysmex, Kobe, Japan) and freshly acquired. Mass cytometry data were analyzed in Cytobank (Beckman Coulter, Brea, CA, USA). Single living cells were determined, and CD45 was used to exclude hematopoietic cells from patient-derived samples. The viSNE analysis (iterations = 2000, perplexity = 30, theta = 0.5), was carried-out on 1 × 10^5^ events. The tSNE-CUDA was carried out on 163,000 cells (iterations = 750, perplexity = 30, theta = 0.5)

### 4.8. Immunohistochemistry

Immunohistochemistry was carried out following standard protocols, as previously described with some modifications [83]. Formalin-fixed, paraffin-embedded tissue blocks were sectioned to 4 μm and mounted on charged glass slides (Superfrost Plus, Thermo Fisher Scientific). The slides were deparaffinized with Bond Dewax Solution (AR9222, Leica, Wetzlar, Germany), rehydrated in descending ethanol solutions to water, and antigen retrieval was performed in the Bond-Max, Leica’s immunohistochemistry platform in Bond Epitope Retrieval 2 solution pH 9.0 (AR9640, Leica, Wetzlar, Germany) with incubation for 20 min. The endogenous peroxidase activity was blocked by incubating in 3% H_2_O_2_ for 5 min followed by washing (Bond Wash Solution, AR9590, Leica). The sections were incubated with the anti-HIF-1α antibody in 1:250 dilution (rabbit monoclonal, EP118 clone, catalogue number: BSB2520; BioSB Santa Barbara, CA, USA) or anti-TMEM45A antibody at 1:800 dilution (catalogue number: orb357227, rabbit polyclonal, Byorbit, Cambridge, UK) at room temperature for 20 min followed by washing of the sections in Bond Wash Solution. Labeling system (Bond Polymer Refine Detection, DS9800, Leica) containing anti-rabbit seconder antibody labeled with horseradish peroxidase (HRP), and DAB-3 (3′-diaminobenzidine) was used as the chromogen for antigen signal detection. Hematoxylin (ready to use, Leica) was used for contrast staining. The Zeiss Axio Imager Z1 microscope (ocular 10×, objectives 10×, 40×) was used for visualization with the Zeiss AxioCam MRm camera and AxioVision SE64 4.9.1 software (Carl Zeiss AG, Oberkochen, Germany).

### 4.9. Statistical Analysis

Statistical analysis was performed using GraphPad Prism 6 (San Diego, CA, USA) and Microsoft Excel (Redmond, WA, USA). Paired *t*-testing was performed between two groups, as indicated in the figure legends. Data are expressed as arithmetic mean ± standard deviation (SD).

## 5. Conclusions

The cell lines used in our study—A549, H1975, and H1650—are the laboratory models most frequently used to study NSCLC and for the development of drugs to treat lung cancer. Our data showed that A549, H1975, and H1650 NSCLC adenocarcinoma cells bear intrinsic single-cell heterogeneity due to the expression patterns of the 13 investigated markers GLUT1, MCT4, CA9, TMEM45A, CD66, CD274, CD24, CD326, pan-keratin, TRA-1-60, Gal-3, Gal-1, and EGFR, which could be used to determine intra- and inter-cell-line heterogeneity. The qRT-PCR and CyTOF analysis revealed that hypoxic microenvironments and altered metabolism influenced the occurrence of single-cell heterogeneity. The marker set was extended to include HLA-ABC, the expression of which was also subsequently investigated in human lung-cancer-derived specimens. We showed differential expression of GLUT1, MCT4, CA9, TMEM45A, CD66, CD274, CD24, CD326, and pan-keratin in the primary lung human adenocarcinoma tissue compared with tissue from the non-involved area. Intra- and inter-cell-line single-cell heterogeneity of A549, H1975, and H1650 cells, as well as ITH of human primary lung adenocarcinoma for the expression of the investigated markers was demonstrated by high-dimensional visualization of stochastic neighbor embedding (viSNE) and FlowSOM analyses. Additionally, our results are the first to show TMEM45A expression in human lung adenocarcinoma, which was verified by immunohistochemistry.

## Figures and Tables

**Table 1:** Antibodies used for mass cytometry.

Catalogue Number	Supplier	Target	Metal Tag
311402	Biolegend	HLA-ABC	^112^Cd
311402	Biolegend	HLA-ABC	^114^Cd
3144017B	Fluidigm	HLA-ABC	^144^Nd
3141006B	Fluidigm	CD326 (EpCam)	^141^Pr
3148012B	Fluidigm	TRA-1-60	^148^Nd
3149018B	Fluidigm	CD66-a/c/e	^149^Sm
3156026B	Fluidigm	CD274 (PD-L1)	^156^Gd
3162027A	Fluidigm	pan-keratin	^162^Dy
3166007B	Fluidigm	CD24	^166^Er
3170009B	Fluidigm	EGFR	^170^Er
3153026B	Fluidigm	galectin-3 (Gal-3)	^153^Eu
3089003B	Fluidigm	CD45	^89^Y
MAB2188-100	R&D Systems	CA9	^158^Gd
MAB1418	R&D Systems	GLUT1	^154^Sm
sc-376140	Santa Cruz Biotech.	MCT4	^171^Yb
orb357227	Biorbyt	TMEM45A	^169^TM
2C1/6	Monostori’s laboratory [33,82]	galectin-1 (Gal-1)	^175^Lu

## Data Availability

The data presented in this study are available on request from the corresponding author.

## Supplementary Materials

The following are available online at https://www.mdpi.com/article/10.3390/cancers14010144/s1. Supplementary Tables S1–S3: The STR profile of A549, H1975, or H1650 cells. Supplementary Table S4: Primers used in the study. Supplementary Table S5: The clinical parameters of human subjects involved in the study. Supplementary Figure S1: Viability of A549, H1975, and H1650 cells after DEF treatment. Supplementary Figure S2: qRT-PCR profile of A549, H1975, and H1650 cells under hypoxic condition. Supplementary Figure S3: Manual gating reveals co-expression of certain markers contributing to ITH of human primary lung adenocarcinoma. Supplementary Figure S4: The expression of TMEM45A with HIF-1α in human lung adenocarcinoma.
