# Supplementary material for: Analysis of the Single-Cell Heterogeneity of Adenocarcinoma Cell Lines and the Investigation of Intratumor Heterogeneity Reveals the Expression of Transmembrane Protein 45A (TMEM45A) in Lung Adenocarcinoma Cancer Patients
Source: Cancers (Basel). 2021 Dec 29;14(1):144. doi: 10.3390/cancers14010144 (PMC8750076; doi:10.3390/cancers14010144)
Supplement: Supplementary file 1 [file cancers-14-00144-s001.zip › Supplementary Figure Legend.pdf]

Supplementary Figure S1. Viability of A548, H1975 or H1650 cells after DEF treatment. The sublethal dose of DEF was determined on A549, H1976 or H1650 cells treated with the serial dilutions (31.25 μM, 62.5 μM, 125 μM, 250 μM, 500 μM, 1000 μM) of DEF for 24h, 48h, or 72h. Viability was measured by the fluorescent resazurin assay as describe in the Materials and Methods section. Red arrow indicates the applied 400 μM for subsequent experiments.

Supplementary Figure S2. The qRT-PCR profile of (A) A549, (B) H1975, and (C) H1650 cells under hypoxic condition after 24h treatment with DEF. Mimicking hypoxia by DEF treatment was well-established because HIF-1α dependent gene expressions were induced except PLAUR and CA9 in H1975 cells and PDGFB, CXCL12, LAMP2 in H1650 cells. The extent of the expression of genes induced by hypoxia showed a characteristic profile for all cell lines under investigation. In line with the literature data TMEM45A mRNA expression was induced upon hypoxia in the studied cells. Relative expression of the analyzed genes was normalized to the mean value of the ACTB reference gene and gene expression changes were calculated from three replicates. Data are expressed as ΔΔCt ($\log_2$) values and were normalized to expression values from cells maintained in normoxia as described in the Materials and Methods section. All values were presented as mean ± standard deviation (SD).

Supplementary Figure S3**.** Manual gating revealed co-expression of certain markers contributing to ITH of human primary lung adenocarcinoma. Cells, singlets and CD45 negative cells were gated in Cytobank (**A**). Gating strategy is shown for the determination of Co-expression of markers gated within CD45- singlets, event counts are demonstrated on the dot plots (**B**-**F**). Fifteen sub-populations were quantified such as: (**B**) CD326+/Pan-Keratin; CD326+/GLUT1+; CD326+/MCT4+; CD326+/Gal-3+; CD326+/CD66+; (**C**) CD66+/Pan-Keratin+; CD66+/GLUT1+; CD66+/MCT4+; CD66+/Gal-3+; (**D**) Gal-3+/Pan-Keratin+; Gal-3+/ GLUT1+; Gal-3+/MCT4+; (**E**) MCT4+/Pan-Keratin+; MCT4+/GLUT1+; (**F**) GLUT1+/Pan-Keratin+. Gating was performed in Cytobank (Beckman Coulter, USA)

Supplementary Figure S4. The expression of TMEM45A and HIF-1α in human adenocarcinoma showed by immunohistochemistry of lung adenocarcinoma patients. All cases showed high (+++) TMEM45A expression and sporadic expression of HIF-1α (+) in the same regions of consecutive sections of the tumor tissues. Therefore, TMEM45A production should not be induced exclusively by hypoxia. Higher magnification (40x objective) was focused within the presented lower magnification (10x objective) of the region of interest (red box). Scale bar is 100 μm.
