# Supplementary material for: Analysis of the Single-Cell Heterogeneity of Adenocarcinoma Cell Lines and the Investigation of Intratumor Heterogeneity Reveals the Expression of Transmembrane Protein 45A (TMEM45A) in Lung Adenocarcinoma Cancer Patients
Source: Cancers (Basel). 2021 Dec 29;14(1):144. doi: 10.3390/cancers14010144 (PMC8750076; doi:10.3390/cancers14010144)
Supplement: Supplementary file 1 [file cancers-14-00144-s001.zip › Supplementary Table Legend.pdf]

Supplementary Table Legend

Supplementary Table S1: The STR profile of A549 cells. Profiling of the human cell line A549 was done by Mycrosynth AG (Balgach Switherland) using highly polymorphic short tandem repeat loci (STRs). STR loci were amplified using the PowerPlex® 16 HS System (Promega). Fragment analysis was done on an ABI3730xl (Life Technologies) and the resulting data were analyzed with GeneMarker HID software (Softgenetics). The analyzed data of the submitted sample matched 100 % to the DNA profile of the cell line A549 (ATCC® CRM-CCL-185™) and 100 % over all 15 autosomal STRs to Microsynth's reference DNA profile of A549 (Mic_150733).

Supplementary Table S2: The STR profile of H1975 cells. Profiling of the human cell line H1975 was done by Mycrosynth AG (Balgach Switherland) using highly polymorphic short tandem repeat loci (STRs). STR loci were amplified using the PowerPlex® 16 HS System (Promega). Fragment analysis was done on an ABI3730xl (Life Technologies) and the resulting data were analyzed with GeneMarker HID software (Softgenetics). The analyzed data of the submitted sample match 96.7 % to the DNA profile of the cell line NCI-H1975 (Cellosaurus, RRID:CVCL_1511_[Pub_200]).

Supplementary Table S3: The STR profile of H1650 cells. Profiling of the human cell line H1650 was done by Mycrosynth AG (Balgach Switherland) using highly polymorphic short tandem repeat loci (STRs). STR loci were amplified using the PowerPlex® 16 HS System (Promega). Fragment analysis was done on an ABI3730xl (Life Technologies) and the resulting data were analyzed with GeneMarker HID software (Softgenetics). The analyzed data of the submitted sample match 100 % to the DNA profile of the cell line NCI-H1650 (ATCC® CRL-5883™) and 100 % over all 15 autosomal STRs to Microsynth's reference DNA profile of NCI-H1650 (Mic_002208).

Supplementary Table S4. Primers used in this study. The symbols of the genes, the names of the coded proteins, sequences of primers, accession numbers, and the sequences of primers used for QRT-PCR reactions are listed.

Supplementary Table S5. The clinical parameters of human subjects involved in the study. The age, gender, tumor diameter, TNM stage (T: size and extent of the primary tumor, N: cancer in the nearby "draining" lymph nodes, M: distant metastasis), and diagnosis of the patients at the time of sampling was registered.
