# Supplementary material for: Analysis of the Single-Cell Heterogeneity of Adenocarcinoma Cell Lines and the Investigation of Intratumor Heterogeneity Reveals the Expression of Transmembrane Protein 45A (TMEM45A) in Lung Adenocarcinoma Cancer Patients
Source: Cancers (Basel). 2021 Dec 29;14(1):144. doi: 10.3390/cancers14010144 (PMC8750076; doi:10.3390/cancers14010144)
Supplement: Supplementary file 1 [file cancers-14-00144-s001.zip › Supplementary Tables S1-S3.pdf]

Supplementary Table S1. The STR profile of A549 cells

Locus	***Chromosomal Location***	***ATCC Marker***	***Customer Sample Typed Alleles***	***Database Alleles***
D3S1358	Chr03		16	16
TH01	Chr11	Yes	8/9.3	8/9.3
D21S11	Chr21		29	29
D18S51	Chr18		14/17	14/17
Penta_E	Chr15		7/11	7/11
D5S818	Chr05	Yes	11	11
D13S317	Chr13	Yes	11	11
D7S820	Chr07	Yes	8/11	8/11
D16S539	Chr16	Yes	11/12	11/12
CSF1PO	Chr05	Yes	10/12	10/12
Penta_D	Chr21		9	9
AMEL	X/Y	Yes	X/Y	X/Y
vWA	Chr12	Yes	14	14
D8S1179	Chr08		13/14	13/14
TPOX	Chr2	Yes	8/11	8/11
FGA	Chr04		23	23

Supplementary Table S2. The STR profile of H1975 cells

Locus	***Chromosomal Location***	***ATCC Marker***	***Customer Sample Typed Alleles***	***Database Alleles***
D3S1358	Chr03		14/15	14/15
TH01	Chr11	Yes	7	7
D21S11	Chr21		28	28
D18S51	Chr18		13	13
Penta_E	Chr15		12/16	12/16
D5S818	Chr05	Yes	11/12	11/12
D13S317	Chr13	Yes	10	10/13
D7S820	Chr07	Yes	8	8
D16S539	Chr16	Yes	12	9/12
CSF1PO	Chr05	Yes	12	12
Penta_D	Chr21		12/13	12/13
AMEL	X/Y	Yes	X	X
vWA	Chr12	Yes	18	18
D8S1179	Chr08		13/16	13/16
TPOX	Chr2	Yes	8/11	8/11
FGA	Chr04		21/24	21/24

Supplementary Table S3. The STR profile of H1650 cells

Locus	***Chromosomal Location***	***ATCC Marker***	***Customer Sample Typed Alleles***	***Database Alleles***
D3S1358	Chr03		18	18
TH01	Chr11	Yes	9.3	9.3
D21S11	Chr21		30	30
D18S51	Chr18		10	10
Penta_E	Chr15		12	12
D5S818	Chr05	Yes	11	11
D13S317	Chr13	Yes	11	11
D7S820	Chr07	Yes	8/9	8/9
D16S539	Chr16	Yes	11/12	11/12
CSF1PO	Chr05	Yes	11	11
Penta_D	Chr21		8	8
AMEL	X/Y	Yes	X	X
vWA	Chr12	Yes	18	18
D8S1179	Chr08		12	12
TPOX	Chr2	Yes	11	11
FGA	Chr04		20	20
