# Supplementary figures and images for: Analysis of the Single-Cell Heterogeneity of Adenocarcinoma Cell Lines and the Investigation of Intratumor Heterogeneity Reveals the Expression of Transmembrane Protein 45A (TMEM45A) in Lung Adenocarcinoma Cancer Patients
Source: Cancers (Basel). 2021 Dec 29;14(1):144. doi: 10.3390/cancers14010144 (PMC8750076; doi:10.3390/cancers14010144)

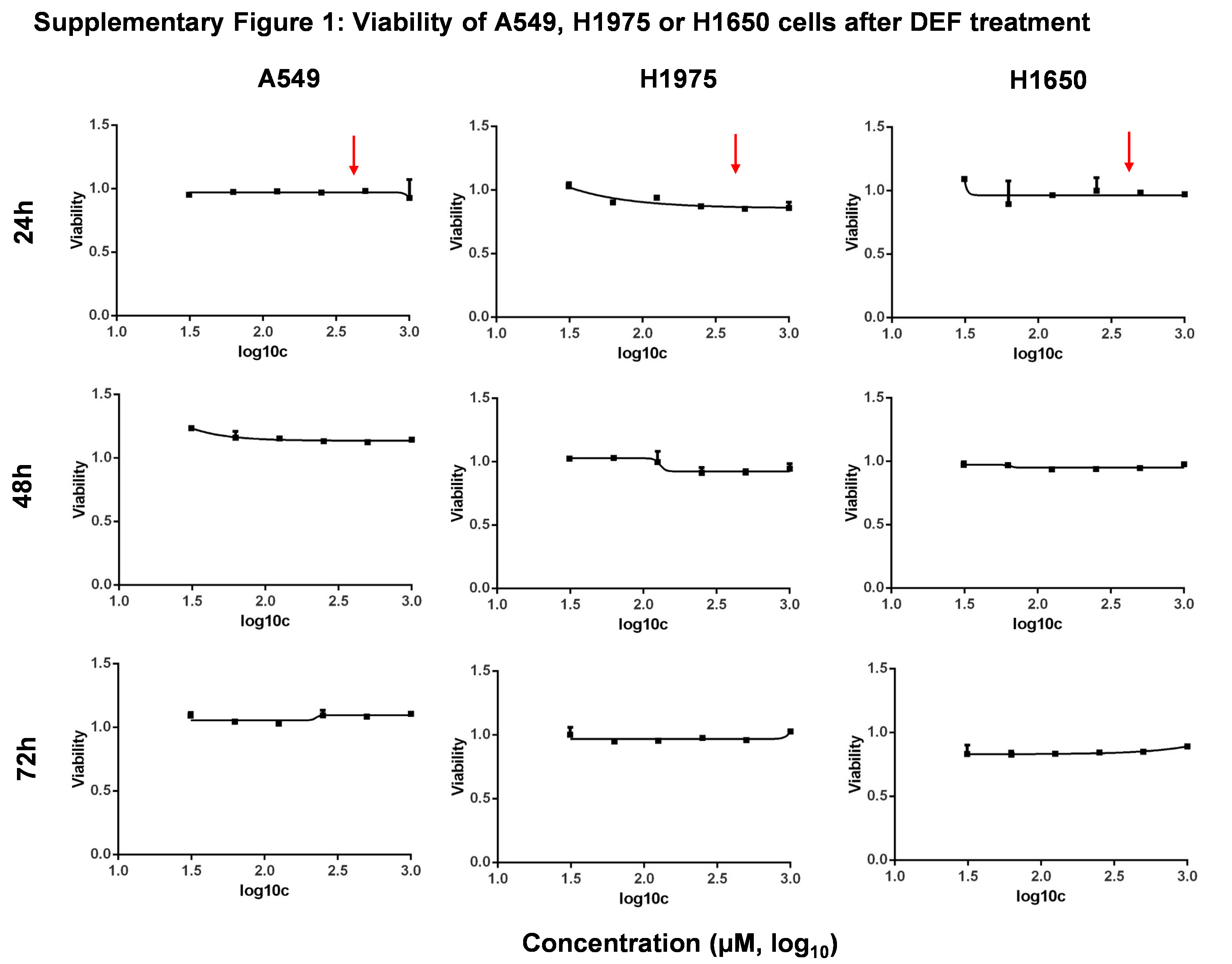

Supplement: Supplementary file 1 [file cancers-14-00144-s001.zip › Supplementary Figure S1.tif]

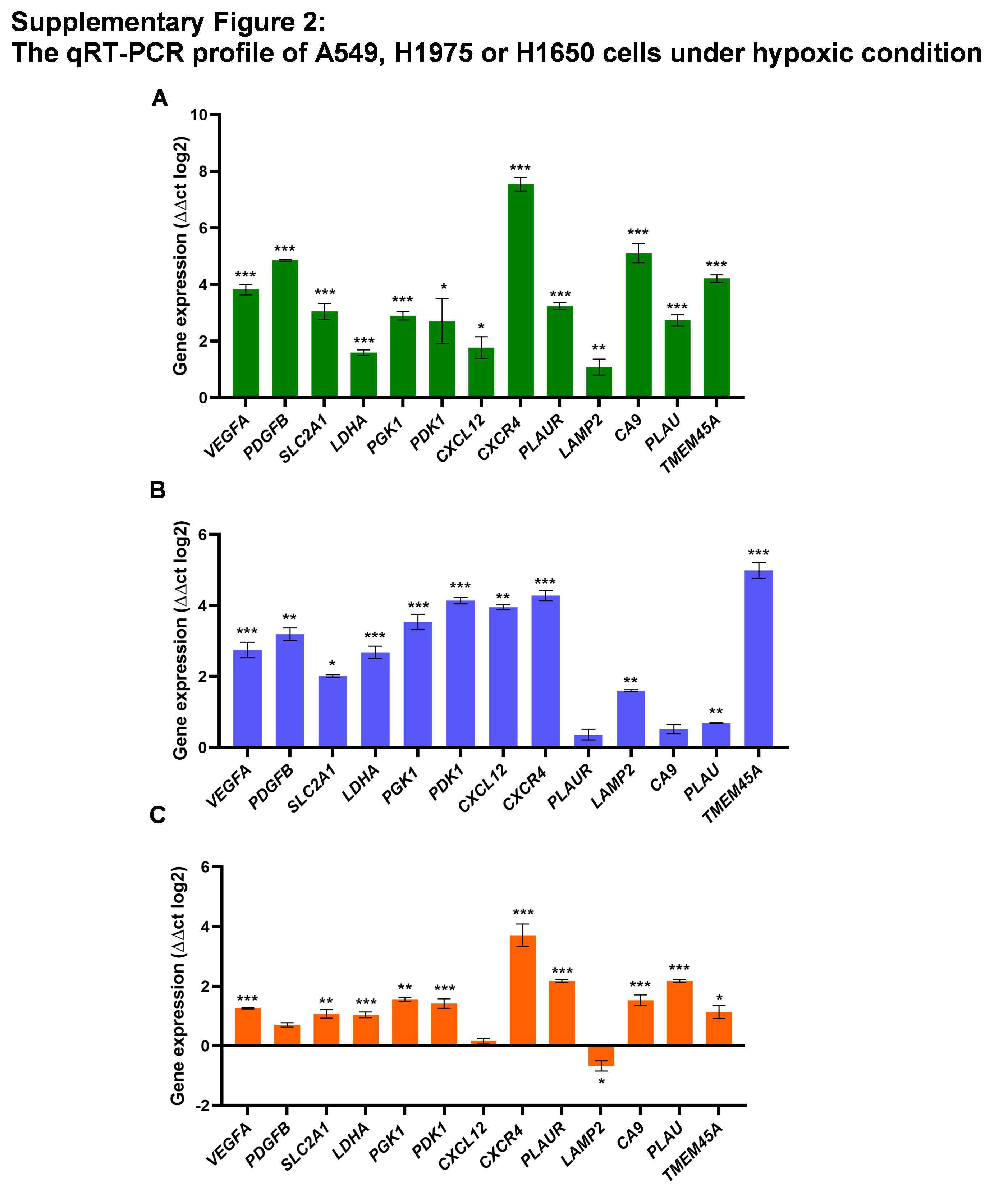

Supplement: Supplementary file 1 [file cancers-14-00144-s001.zip › Supplementary Figure S2.tif]

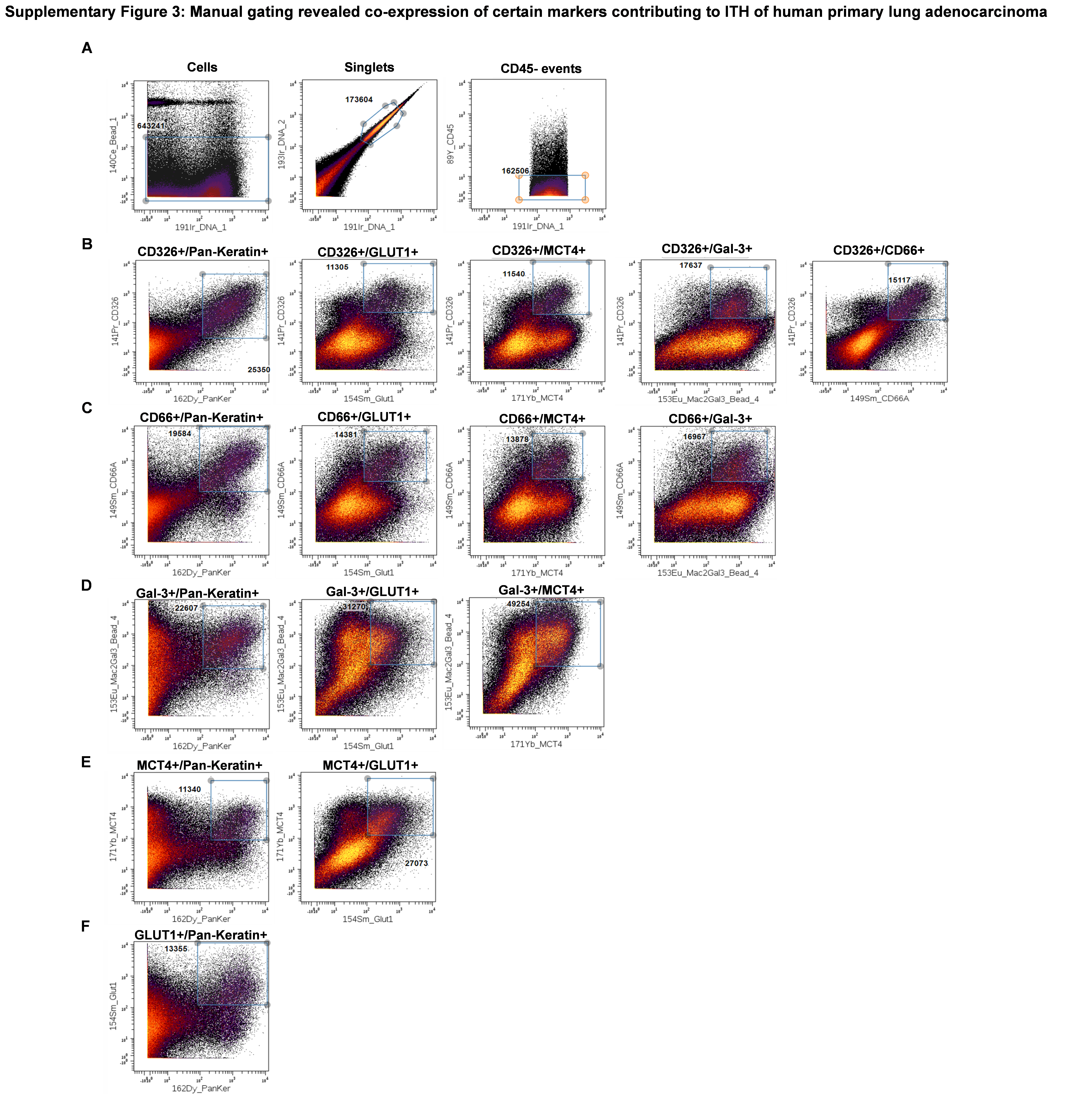

Supplement: Supplementary file 1 [file cancers-14-00144-s001.zip › Supplementary Figure S3.tif]
